# A Comparative Analysis of the Magnetization Methods Used in the Magnetic Nondestructive Testing of Reinforced Concrete Structures

**DOI:** 10.3390/ma16217020

**Published:** 2023-11-02

**Authors:** Paweł Karol Frankowski, Tomasz Chady

**Affiliations:** Faculty of Electrical Engineering, West Pomeranian University of Technology in Szczecin, ul. Sikorskigo 37, 70-313 Szczecin, Poland

**Keywords:** magnetization, nondestructive testing (NDT), simulations, nondestructive evaluation (NDE), reinforcement bars detection, reinforced concrete, rebars, concrete inspection

## Abstract

This work presents how significantly the proper selection of the magnetization method can improve almost all parameters of the magnetic method and affect the effectiveness of the evaluation of reinforced concrete (RC) structures. Three magnetization methods are considered in this paper: opposite pole magnetization (typical solution), same pole magnetization, and (as a reference point) no magnetization. The experiments are carried out in a three-dimensional (XYZ) space. Measurements along each of the axes are discussed in a separate section. The results show that the appropriate selection of the magnetization method can affect noise reduction, signal strength, and the separation of measurements carried out on different samples. This paper also discusses the situations when the magnetization may change the shape, cause deformations of waveforms, affect the area testing, and be used to significantly increase the efficiency of simultaneous evaluation of three basic parameters of RC structure. Experiments and simulations have proven that properly applied magnetization may strongly affect the evaluation’s effectiveness, making the magnetic method one of the most promising techniques in testing RC constructions.

## 1. Introduction

Evaluation of the condition of reinforced concrete (RC) structures by nondestructive testing (NDT) methods is of great practical importance. The domination of RC material in the construction industry has been established worldwide for over a hundred years. Usually, RC structures are designed for fifty to one hundred years [1] of exploitation time. Numerous constructions built at the beginning of the XX century are still in service [1], and their exploitation time is coming to an end. The most current (multi-billion) issue is the reinforcement corrosion in constructions of this type. However, it is only one of many possible defects [1,2,3].

Periodic inspection of old buildings is (in most countries) required by law. Mostly, inspections of this kind should be conducted once per five years. On the other hand, newly built facilities require acceptance tests. Such tests are performed to verify if the requirements of a specification or contract are met. Such inspections should be executed after the concrete has hardened. It is also important not to damage the newly built structure during the investigation. For all these reasons, it is necessary to develop and adopt NDT methods for RC construction diagnostics [1,4,5,6,7]. One of the NDT methods that can face all these problems is the magnetic method. However, the magnetic tests’ efficacy greatly depends on the level of magnetization of the reinforcing bars, which is unknown. Therefore, a proper excitation system can eliminate unknowns, significantly improve all method parameters, and simplify parameter identification [6,7,8].

### 1.1. Magnetic Methods

Magnetic techniques are a considerable group of various methods, which can be split into two groups: continuous magnetization techniques (CMT) and residual magnetization techniques (RMT). The CMT uses a magnet or electromagnet-based excitation system. The RMT is passive. There are many applications of magnetic methods in the construction industry. These methods can be used to detect reinforcement and determine the parameters of RC structures [9,10,11,12] or to detect corrosion of rebars [13,14,15,16,17], cracks or defects [18,19], and rebar stress [20,21,22]. A more detailed description of the applications of magnetic methods in the construction industry can be found in [6,22].

The magnetic flux leakage (MFL) technique is primarily representative of CMT. The propagation of magnetic flux may be disturbed by large inhomogeneities like rebar (in concrete walls) or more minor discontinuities on the rebar (breaks, cracks, or caused by corrosion diameter reduction) [6,23]. Other active magnetic methods, such as magnetic powder, stress-induced magnetic anisotropy, magneto-acoustic, or Barkhausen, are usually not applied to assess RC constructions.

The RMT can detect unusual conditions coming from changes in the structure of the crystalline (caused by concentration, stress, cracks, or corrosion) [24,25]. The most typical representation of this group in civil engineering is the Magnetic Memory Method (MMM). MMM can come in various variants. One of them is the Infrastructure Corrosion Assessment Magnetic Method (iCAMM). The technique is a passive magnetic inspection working under the effect of Earth’s magnetic field [16,19]. A comparison between RMT and CMT is shown in Table 1.

The most commonly used magnetic sensors are magnetoresistance (MR) and Hall effect elements (98% of the market of magnetic sensors) [26,27]. Very small size (in the order of micrometers), low power consumption, and the compatibility of Hall and MR sensors with CMOS technology enable placing three sensors in one housing. Such constructed elements can measure *B*_x_, *B*_y_, and *B*_z_—all three spatial components of magnetic induction. The MR, opposite to Hall effect sensors, can be varied in an extensive range (sensitivity). The other advantages are a considerably large exchange bias field, narrow hysteresis, and minor changes of parameters with temperature, sensitivity, reliability, and repeatability. Theoretically, all of these parameters are better in the case of GMR than AMR elements and much better than in the case of Hall effect elements. Hall sensors are cheaper, less sensitive, and used for measuring industrial-level magnetic fields. However, AMR technology offers higher bandwidth and measurement of negative values (polarization). Sensors of this kind also have a reset system that restores them to their original state after exceeding the measurement range [7,8,20,27,28,29]. The high usefulness of the possibility of examining the spatial components of the magnetic field induction was proved in [8].

The significant advantage of the magnetic technique is that it is one of the few NDT methods that allow conducting area tests of reinforced concrete constructions in a cheap, fast, and straightforward way. The method can be used for the preliminary finding of rebars in large-sized constructions (e.g., using a magneto-optical MO sensor or matrix of MR sensors) [7,8].

### 1.2. Magnetic Method in Comparison to Other Nondestructive Tests

A review of NDT techniques utilized in RC construction diagnostics (with their advantages and disadvantages) is provided in [4,5,6,7,8,9]. Several properties of structures of this kind can be tested with NDT methods, including the strength of concrete, visual condition, carbonation level, electromagnetic properties, mechanical properties, thermal properties, and natural frequencies [7]. Most of these NDT techniques are intended to test concrete. Only electromagnetic and mechanical wave systems can be efficiently applied for direct reinforcement evaluation [6,7].

The mechanical wave techniques are universal and can be used to test concrete and rebars. On the other hand, many factors influence the mechanical methods’ results. Various phenomena may disturb the propagation of mechanical waves in complex structures. A mechanical wave is also significantly damped by the concrete. Therefore, in many cases, electromagnetic and magnetic techniques are favored to evaluate reinforcement elements in concrete constructions [6,7].

Magnetic and electromagnetic waves mainly impact rebars. For such waves, concrete is nearly transparent. Therefore, these methods may be very effective and precise [6,7,8]. Among the NDT methods based on an AC magnetic field, the eddy current (EC) technique is the most important. The EC tests can be utilized to detect steel bars in RC construction and accurately estimate the value of the main parameters of RC construction (rebar’s diameter, rebar class, and thickness of the concrete cover [30,31,32,33,34]). Under some conditions, the method can also identify corrosion or other flaws [35].

The magnetic evaluation is utilized for similar functions to the EC technique. However, magnetic sensors can be used for area testing. Such investigation with EC evaluation would be relatively challenging. The next advantage of the magnetic method lies in a simple excitation system. This element makes the magnetic method very applicable and inexpensive. The other massive advantage is the possibility of analyzing specific spatial magnetic components [7,8].

The highest limitation of DC magnetic tests compared to EC testing is spatial resolution. The utilized excitation frequency can categorize electromagnetic (and mechanical) NDT techniques. The same technique may have a good effective range and low resolution in the case of low-frequency excitation or a good resolution and limited range in the case of high-frequency excitation. (The fundamental division of NDT techniques due to the excitation frequency is delivered in [6,7,8].) The spatial resolution and sensitivity of the EC method can be adjusted by adjusting the frequency. The magnetic method does not provide such a possibility. In evaluating RC structures, the resolution of magnetic tests is lower than in the case of the EC method. However, this issue is of little importance for inhomogeneities as large as reinforcing bars in concrete.

The capacitive method is the third group of techniques with a potential similar to EC and magnetic methods. This technique works in a frequency range similar to the eddy current tests (frequencies of 10^2^ Hz to 10^9^ Hz). The effective range of the capacitive method is also smaller than that of the EC or magnetic techniques. However, the method enables the detection of smaller inhomogeneities [36,37,38]. The comparison of magnetic, capacitive, and EC methods is presented in Table 2.

In addition to the magnetic and capacitive methods, a few other electromagnetic methods are well fit to quickly evaluate large areas of the RC structure. However, each of them has significant disadvantages:

1. Ground-penetrating radar (GPR) and other microwave methods have many advantages. The ground-penetrating radar is an up-and-coming method. Access to only one side of the wall is required. Rebars can be detected from several centimeters up to over ten meters (other magnetic or electromagnetic methods usually have a maximum detection range below 200 mm). Under some conditions, the GPR can be used to estimate the rebars’ diameter and detect defects, breaks, or even debonding caused by corrosion. The method may also be applied to mapping multilayer reinforced meshes. Unfortunately, the method also has several disadvantages. Similar to mechanical wave methods, many factors like variable internal moisture conditions or voids may affect the results. The GPR device is expensive. The systems of this kind are not fitted well to low concrete cover thickness. The next problem lies in problems with identification caused by difficulties with results interpretation and limited resolution [6,39,40,41,42,43,44];
2. Infrared thermography (IR) is usually utilized in order to examine concrete. The method is also one of the very few that can be applied for the preliminary detection of rebars in large-sized constructions (area testing) [6] and sometimes (under many conditions) to detect corrosion. The method’s effectiveness is related to the concrete cover thickness. The method can be successfully applied when the cover thickness is below 50 mm. Moreover, the method required heating and cooling-down phases, which makes it time-consuming. Therefore, it is not commonly used in practice [6,30,45,46,47,48,49,50];
3. Radiography is a very effective method. The range and resolution are very high. The method can easily be used for the evaluation of RC structures. However, there are several reasons why this method is rarely used for this purpose. Tests of this kind can generate risks to human health. Usually, access to both sides of the examined object is required, measurements are not that fast, and the devices are expensive [6].

### 1.3. Motivation

The concept for this paper arose during the investigations on increasing the efficiency of the M5 system [6] through sample magnetization. During this research, it turned out that the magnetic method’s effectiveness is high with proper magnetization. Moreover, the technique has many advantages over competing techniques (this issue is explored further in this article). Therefore, the research was significantly expanded. The first paper in this series [7] focuses on the potential of the magnetic method for rapid testing of a large area (area testing). The work tested the potentials of Anisotropic Magneto Resistance (AMR) and magneto-optical (MO) sensors for such tests. The second paper [8] delved into the problem of efficiency and simplicity of RC structure parameter simultaneous identification. It was indicated that not advanced and sophisticated classifiers but properly selected (in small numbers) and independent (from each other) attributes are fundamental. In order to obtain independence, accurately reflect the waveform shape, and reduce the number of attributes straightforwardly, the ACO (from Amplitude, Correlation, and Offset) decomposition was proposed. The effectiveness of the ACO method was proved. The problem of identification in situations where it is complicated to collect an adequately extensive database has also been raised.

This work aims to show the influence of magnetization on the efficiency and quality of magnetic measurements. This paper also pointed out how to implement and utilize magnetization to improve signal strength signal-to-noise ratio, use magnetization in area testing, or separate attributes to improve classification efficiency. According to the authors, the issue of magnetization is generally underestimated and insufficiently explored. Very few works are connected with rebar magnetization [7,51,52], and also not many about magnetization in the magnetic method generally. The presented results can be easily interpolated to magnetic tests of other types (including identification of parameters and detection of inhomogeneities or defects in other structures) or be a starting point for improving the performance of some electromagnetic methods like M5 [6] or eddy current.

## 2. Materials and Methods

This section provides technical details about the measurements, the measurement system, and the methods used in the research.

### 2.1. Samples

**Figure 1:**
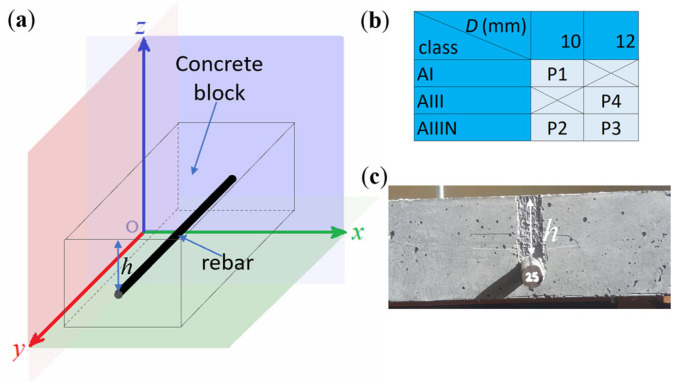
Example of the sample and sample parameters: (**a**) presentation of an example sample in the coordinate system, (**b**) parameters of the rebars used in the experiments, (**c**) example of the RC sample (number 25 on the rebar is the sample number).

The RC structures are represented by three basic parameters: rebar diameter *D*, rebar class, and concrete cover thickness *h*. Typical for RC constructions, *h* ranges from twenty to fifty millimeters. This range has been extended in the research (relative to construction standards) and is equal from twenty to seventy millimeters, with the step of ten millimeters (*h*_20_, *h*_30_, *h*_40_, *h*_50_, *h*_60_, *h*_70_). In the tests, rebars with diameters of *D* = 10 mm and *D* = 12 mm are used. The same elements are frequently utilized in practice. The used rebars are made of three popular classes of steel: AI (highest flexibility and lowest hardness of the alloy), AIII (low flexibility and high hardness), and AIIIN (lowest flexibility and highest hardness). The classes are marked according to the national standard [53]. The parameters of the used samples are presented in Figure 1. (Since concrete does not affect the results of magnetic measurements, its description is omitted.)

Four kinds of rebars are utilized in the tests: P1: *D*_10_-AI, P2: *D*_10_-AIIIN, P3: *D*_12_-AIIIN, and P4: *D*_12_-AIII. These steel bars are available at most building supply stores. The widespread use of these types of bars in the construction industry makes the experiments relevant to civil engineering reality. Standardization of reinforcing bars is carried out solely for mechanical properties. The same reinforcement elements received from different manufacturers may have different magnetic properties. A solution to this problem was proposed in [8].

### 2.2. Measuring System and Measurements

**Figure 2:**
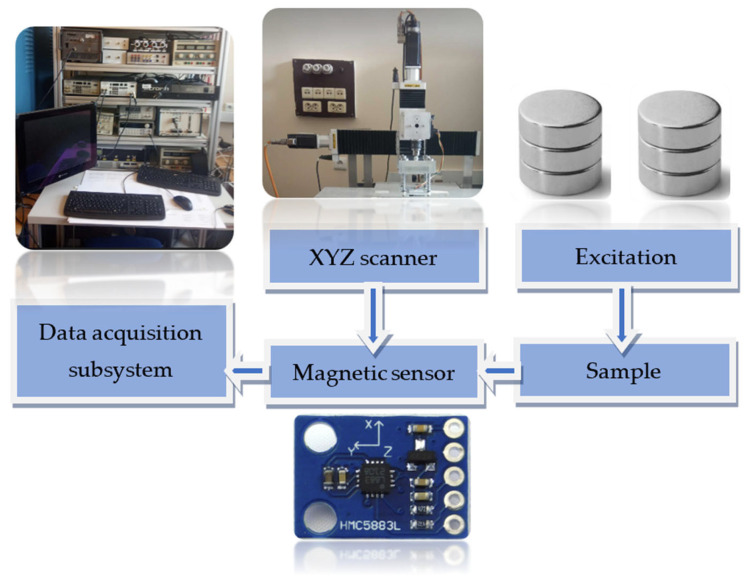
Block scheme of the measuring system.

The designed measuring system is very straightforward and consists of five elements: excitation subsystem, positioning subsystem, tested sample, magnetic transducer, and data acquisition subsystem. The block diagram of the used magnetic system is presented in Figure 2.

**Figure 3:**
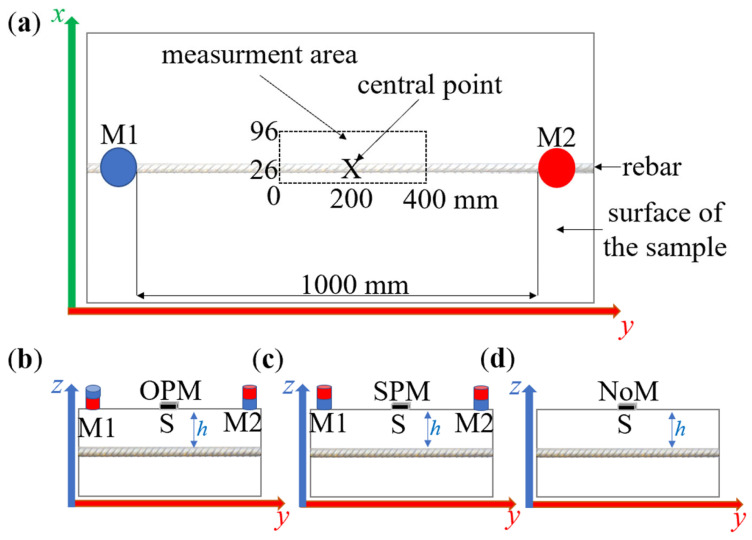
Schematic view of the sample with depicted measurement area, where M1 and M2—magnets; S—sensor (HMC5883L). (**a**) 2D view from the top; (**b**) 2D side view—OPM; (**c**) 2D side view—SPM; (**d**) 2D side view—NoM.

The excitation subsystem (Figure 3a–c) consists of two neodymium magnets, M1 and M2, placed on the sample’s surface, directly above the rebar (between the rebar and magnets is a concrete cover with a thickness of *h*). Two different magnet configurations are tested in this work. The first arrangement is opposite pole magnetization (OPM). In this case, the magnets have opposite poles facing the sample (Figure 3b). The other one is the same pole magnetization (SPM). The magnets are orientated to the sample with the same poles (Figure 3c). As a reference point, the case NoM without magnetization is also tested (Figure 3d). The magnets are not moved during the measurements. The distance between M1 and M2 equals 1000 mm and was determined experimentally. Magnets should be strong enough to magnetize the rebar from the distance *h*. However, magnets that are too strong too strong can affect the sensor directly and strongly deform the results.

The samples are tested with an HMC5883L sensor (HMC5883L, Honeywell, Morris Plains, NJ, USA). This AMR sensor (S) can measure spatial components of magnetic induction (*B_x_*, *B*_y_, and *B*_z_) and has high sensitivity and field resolution. The measurement range is from −0.8 to 0.8 T. The transducer moves over the concrete’s surface in two orthogonal directions, *x* and *y*. Small size, relatively low power consumption, and compatibility with CMOS technology enable the assembly of a matrix of HMC5883L sensors to build an area-testing transducer.

Reinforcing bars from ten to twelve millimeters in diameter are usually connected into a reinforced grid with 150 or 200 mm (150 × 150 or 200 × 200 mm) eyes. In such a dense mesh, nearby reinforcing bars could significantly distort the measurement results. Therefore, the measurement should be limited to only a few centimeters in front of and behind the tested rebar. Due to the symmetry of the waveforms, the measurements on one side of the reinforcing bar were shortened. It allowed us to shorten the measurement time without significant loss of information. Measurements in the *x*-axis were made in steps of 1 mm in the range of 0 to 96 mm and the *y*-axis with steps of 10 mm (from 0 to 400 mm). The central point (in the middle of the rebar) is *x* = 26 mm, *y* = 200 mm. Measurements made in this way in two planes were recorded and subsequently analyzed.

### 2.3. Methods Used in the Analysis, Signal Processing, and Data Acquisition

#### 2.3.1. Calculation of the SNR

The waveforms presented in this work have not been processed or filtered in any way. The measured constant field distributions are slowly changing in space. The changes in slow-changing waveforms caused by the presence of a rebar are superimposed on disturbances. The interference consists of external changing fields and measurement noise from the sensor systems. The signal-to-noise ratio (SNR) is calculated to compare magnetization methods and determine which is more susceptible to noise [54]. The SNR is provided by Formula (1).

(1) $$\mathrm{SNR}=20\frac{\log(A_{signal})}{\log\left(A_{noise}\right)}$$

where *A_signal_* is the signal’s amplitude, and *A_noise_* is the amplitude of the noise.

**Figure 4:**
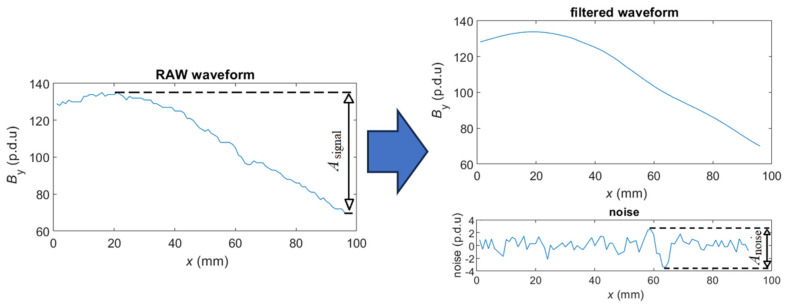
Separation of the RAW waveform into noise and filtered waveform, along with marked amplitudes.

The SNR is calculated by comparing the signal amplitude to the noise amplitude according to Equation (1). The definition of amplitude is presented in Figure 4.

Many different methods can be used to separate noise from signal. Several of them were tested in this research (during SNR calculation). Simple statistical filters such as the moving average filter or the median filter do not produce good results; the error of the method is significant (especially in the case of signals with high SNR). The Savitzky-Golay (S-G) [55,56] filter was well-suited to filter out relatively noisy waveforms. The delay caused by the filter is also not a significant problem in this case. Unfortunately, due to fast changes in the waveform value, the polynomials generated by the S-G algorithm did not perfectly overlap the original waveform of the *B*_x_ component (low *h*). As a result, in the case of smooth waveforms (SNR > 80 dB) the method error was greater than the noise. What could be noticed by the decreases in SNR values at small *h*. The filter can be recommended in cases where the difference between the frequency spectrum of the interference and the signal is small or when the signal value changes very slowly (then the polynomials will fit well). If the frequency spectrum of the noise is in the range of significantly higher frequencies than the signal spectrum, then a Butterworth low-pass filter is a better solution, and this filter was used in the experiments. A more extensive comparison of basic filters for a similar issue was discussed in [27].

#### 2.3.2. Boxplot Graphs

**Figure 5:**
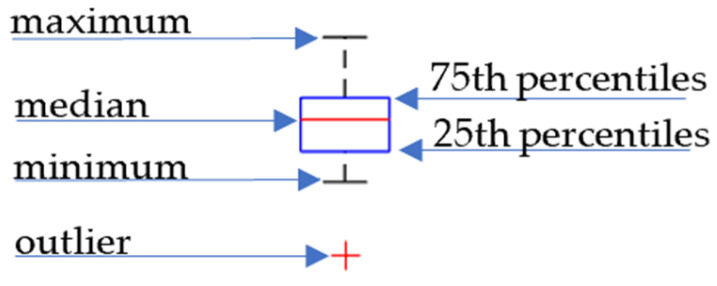
Description of the used boxplot.

In this article, the boxplot is used to present the statistical distribution of the selected variable. The description of the used boxplot is shown in Figure 5.

The red mark in the blue rectangle indicates the median. The bottom edge of the box indicates the 25th percentile. The top one is the 75th percentile. The most extreme data points (excluding outliers) are represented by the whiskers when the outliers are marked as ‘+’ marker.

#### 2.3.3. A Measure of Class Separation Independent of Amplitude

In order to calculate separations between classes, clusters, or points, the Euclidean distance is usually used. This parameter is provided by Formula (2).

(2) $$d_{a}\left(\alpha ,\beta \right)=\sqrt{\sum_{i=1}^{n}{\left(\alpha_{i}-\beta_{i}\right)}^{2}}$$

where α and β are measurements placed in *n*-dimensional space.

However, using the absolute value of Euclidean distance (*d_a_*) to compare the separation between the results obtained by different magnetization methods is suboptimal and may be ineffective. The achieved results are significantly related to the signal amplitude. During the analysis, examined variables should be possible as independent of each other. This issue is discussed in more detail in [8]. In order to make the separation independent of the signal amplitude, Equation (3) was determined. The separation is not provided as an absolute value in this case but as a percentage of the average amplitude of the waveforms. This relative distance (*d*_r_) allows us to compare different magnetization methods.

(3) $$d_{r}\left(\alpha ,\beta \right)=\sqrt{\sum_{i=1}^{n}\frac{{\left(\alpha_{i}-\beta_{i}\right)}^{2}}{{\left(\left(\alpha_{i}+\beta_{i}\right)/2\right)}^{2}}}\times 100\%$$

## 3. Results

According to the obtained results, magnetization has a crucial effect on the results of magnetic evaluation [7]. In this section, the effect of the use of three different magnetization variants, SPM, OPM, and NoM, on results is discussed. Magnetization may impact many factors like noise level, deformation of the waveform shape, amplitude, or even separation between the classes (and as an effect on the identification results). It also can limit the effective measurement range. All these effects are discussed in this section. This part is divided into four subsections. Firstly, the results of numerical simulations are presented. Then, in each of the remaining subsections, the influence of magnetization on measurements in a different *x*, *y*, or *z*-direction (*x*-scan, *y*-scan, or *z*-scan) is presented. Based on the results, it is analyzed how scans in a specific dimension can be used in practice and what benefits result from the use of individual methods of magnetization.

All tests are performed with the same magnets and AMR sensors. Therefore, the impact of the magnetization is weaker for samples with a higher *h*.

### 3.1. Numerical Simulations

The finite element method (FEM) was used for numerical analysis. A commercial COMSOL v6.0 software package was utilized for this purpose. The 3D model with the “Magnetic Fields, No Currents” interface from the AC/DC Module was selected for this analysis. Due to the fact that the magnetic field near the sensor was relatively small and to obtain precise information about its change, the analysis area was divided into a relatively large number (over 2 million) of finite elements. A fine mesh around the magnet and iron rebar was also utilized, as this is where the magnetic field was the strongest. The analysis was carried out for two different orientations of the magnet poles and varying distances between the magnets and the reinforcing bar.

**Figure 6:**
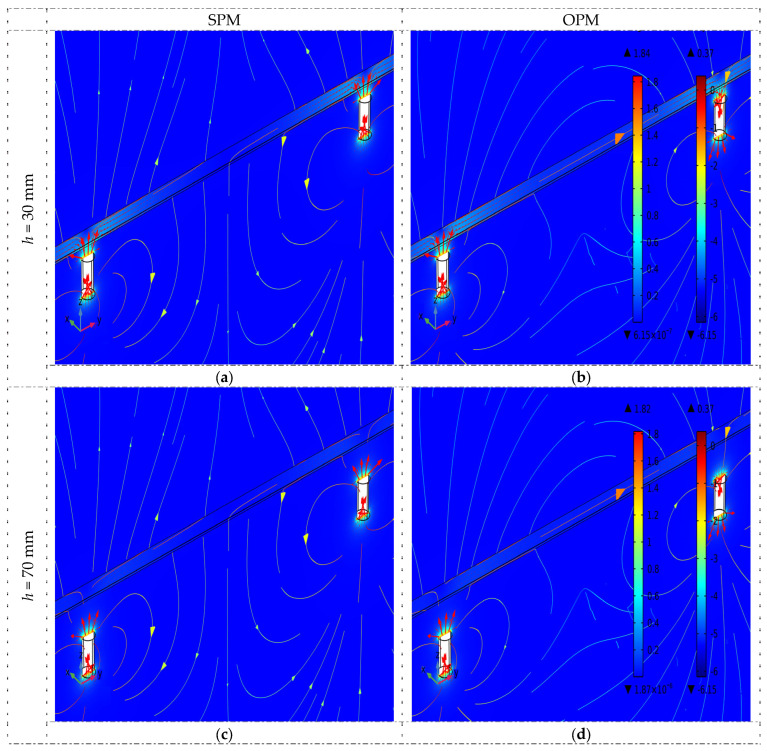
The spatial distribution of normalized magnetic flux density lines received for both methods of magnetization (OPM and SPM) and two concrete cover thicknesses 30 and 70 mm (simulations) and magnetic permeability *µ* = 100. (**a**) SPM, *h* = 30 mm, (**b**) OPM, *h* = 30 mm, (**c**) SPM, *h* = 70 mm, (**d**) OPM, *h* = 70 mm.

The spatial distribution of normalized magnetic flux density lines received for both magnetization methods (OPM and SPM) are shown in Figure 6. This figure’s results indicate that the thickness of the concrete cover is important for the field distribution. The smaller the *h*, the greater the magnetic flux density and the stronger the obtained signal. Two cases are shown in Figure 6: *h* = 30 mm and *h* = 70 mm.

The most minor intensity of the field lines is observed precisely in the middle between the magnets (both SPM and OPM). However, this is also a place where the direct impact of the magnets on the sensor is almost negligible. Therefore, measurements should be taken close to the center but not strictly at it (the measurement area shown in Figure 3). It is also crucial that the measurements are performed in precisely the same place. Otherwise, the amplitudes may be affected by the sensor’s changed position and be incomparable. The waveform’s amplitude changes relatively fast when the sensor moves along the rebar.

**Figure 7:**
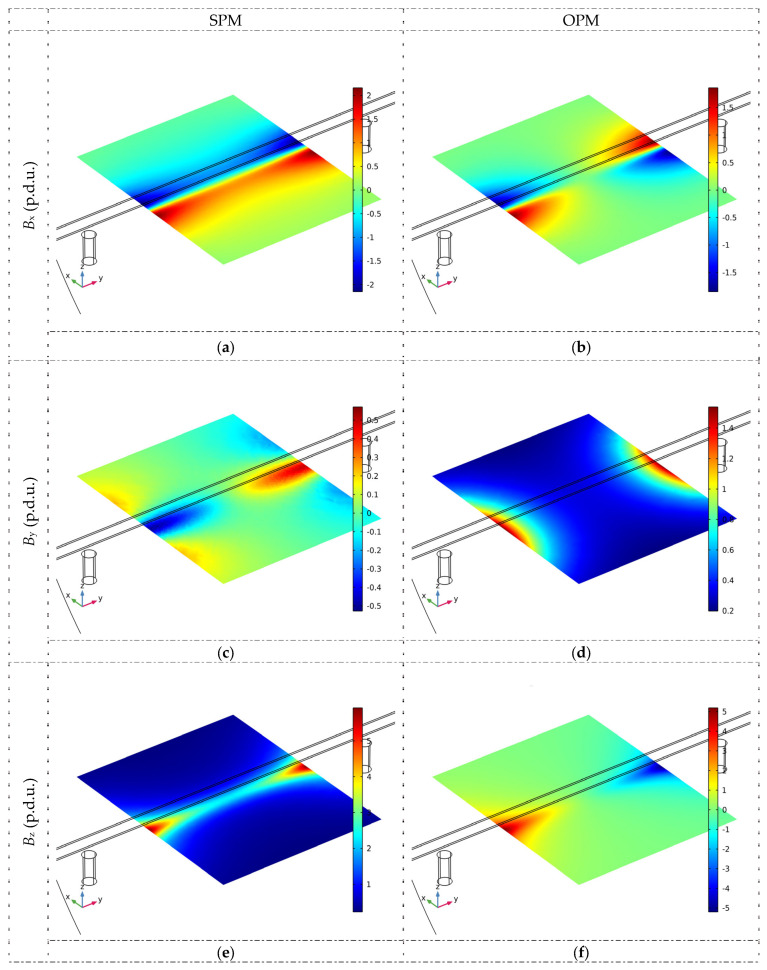
The magnetic flux density distribution in the XY plane for all spatial components and both magnetization methods (simulations), *h* = 30 mm, magnetic permeability *µ* = 100 (**a**) SPM, *B*_x_, (**b**) OPM, *B*_x_, (**c**) SPM, *B*_y_, (**d**) OPM, *B*_y_, (**e**) SPM, *B*_z_, (**f**) OPM, *B*_z_.

The magnetic flux density distribution in the XY plane for all spatial components and both magnetization methods is shown in Figure 7.

**Figure 8:**
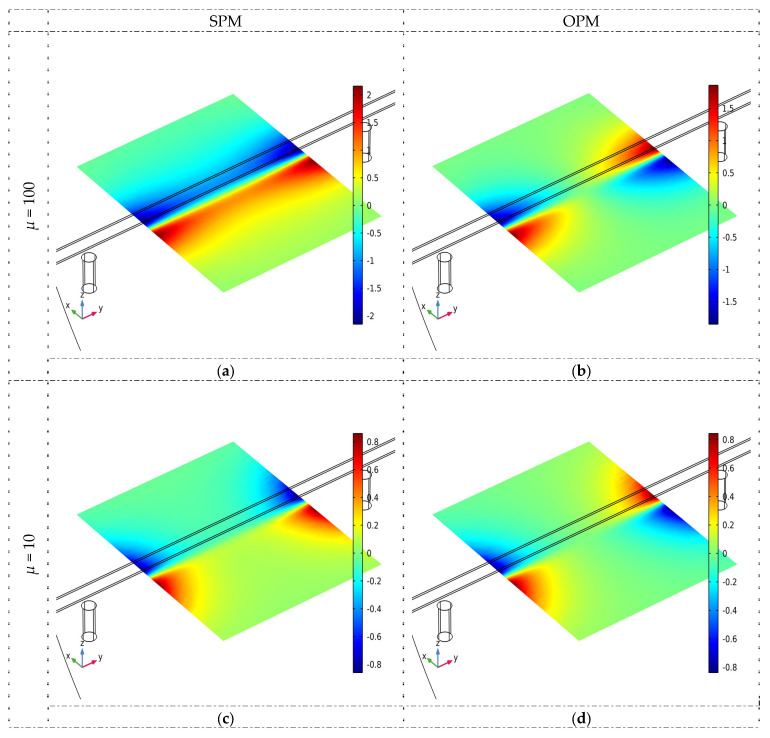

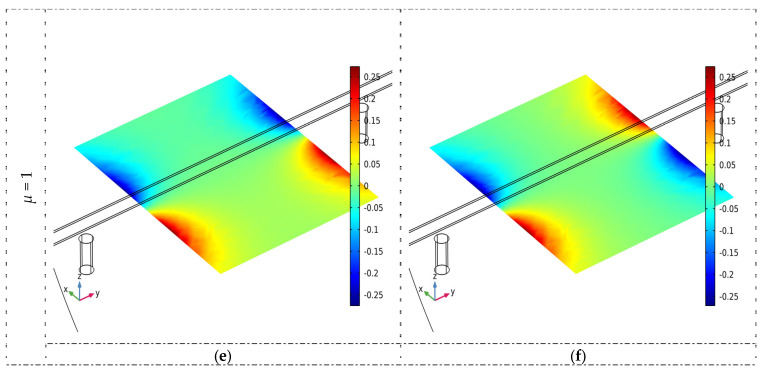
The magnetic flux density distribution in the XY plane for different magnetic permeability (simulations), *B*_x_, and *h* = 30 mm. (**a**) SPM, *µ* = 100 (**b**) OPM, *µ* = 100 (**c**) SPM, *µ* = 10 (**d**) OPM, *µ* = 10 (**e**) SPM, *µ* = 1 (**f**) OPM, *µ* = 1.

Simulations have shown that the tested material’s magnetic permeability (*µ*) can also strongly impact the results. In the case of steel rebars, the greater the *µ*, the higher the magnetic flux density, and the stronger the signal received from the magnetic sensor. Further experiments have shown that, in most cases, the hardness of steel is correlated with its magnetic permeability. Example results of the simulations are shown in Figure 8.

**Figure 9:**
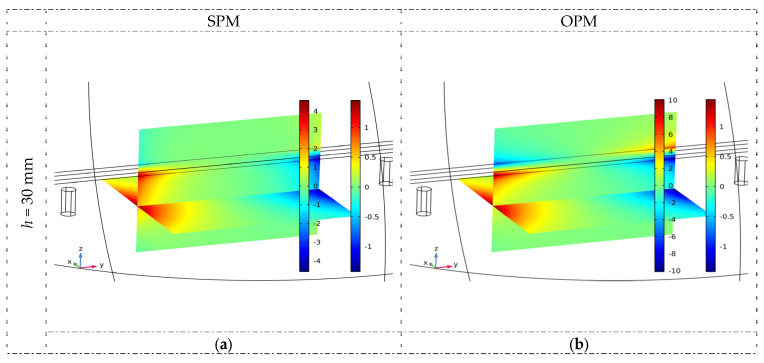

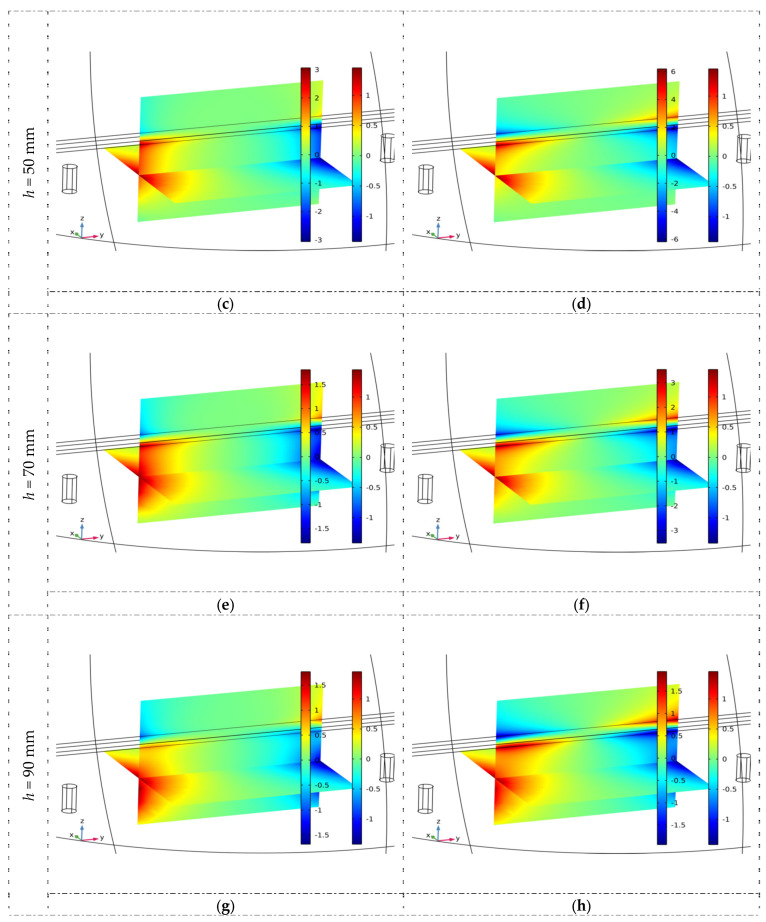
The magnetic flux density distribution in the XY and YZ planes for different magnetic permeability, and *h* (simulations), *B*_z_, OPM (**a**) *h* = 30 mm, *µ* = 10, (**b**) *h* = 30 mm, *µ* = 100, (**c**) *h* = 50 mm, *µ* = 10, (**d**) *h* = 50 mm, *µ* = 100, (**e**) *h* = 70 mm, *µ* = 10, (**f**) *h* = 70 mm, *µ* = 100, (**g**) *h* = 90 mm, *µ* = 10. (**h**) *h* = 90 mm, *µ* = 100.

The impact of the change of *h* and *µ* on the magnetic flux density distribution is presented in Figure 9. The results shown in Figure 9 are projected onto the XY and YZ planes.

**Figure 10:**
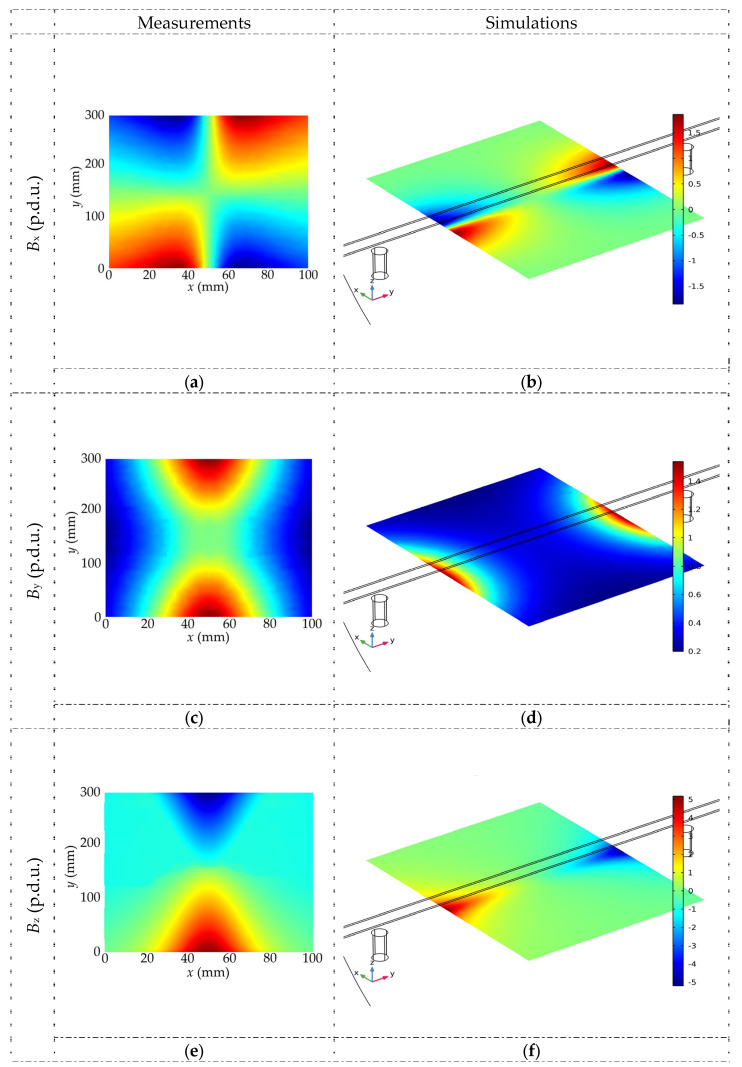
The comparison between actual measurements and simulations; magnetic flux density distribution in the XY plane for all spatial components, OPM, *h* = 30 mm, magnetic permeability *µ* = 100. (**a**) measurement, *B*_x_, (**b**) simulation, *B*_x_, (**c**) measurement, *B*_y_, (**d**) simulation, *B*_y_, (**e**) measurement, *B*_z_, (**f**) simulation, *B*_z_.

Figure 10 shows a comparison between actual measurements and simulations. It can be observed that changes in the magnetic field induction value in the function of position are faster in the case of real measurements than in the developed numerical model. This is due to the lack of complete data on the magnetic properties of rebars. However, the general dependencies are the same, and the results achieved are similar.

### 3.2. Impact of Magnetization on the Measurements Made along x-Axis

The section discusses two important aspects of magnetic testing. The first issue is the impact of the magnetization method on the noise level. The other one is the separation of rebar classes (the ability to recognize the material and the size of inhomogeneities). Usually, rebars are scanned in the *x* direction (*x*-scans). Such scans can be easily used to identify and detect inhomogeneities (defects or the presence of materials such as rebars). Therefore, *x*-scans have been chosen to test noise level and class separation.

#### 3.2.1. Impact of Magnetization on the Amplitude and Noise Level

Reducing the noise level is one of the main advantages of magnetization in magnetic evaluation. Superiorities of CMT (continuous magnetization techniques) over RMT (residual magnetization techniques) in this area were discussed many times, e.g., in [7]. This subsection includes an analysis of to what extent the use of magnetization can reduce noise. It also considers how effective different types of magnetization (OPM, SPM) may be and how these methods affect individual spatial components in the cases of different concrete cover thicknesses (*h*) and different reinforcing bars. In order to correctly estimate the significance of the noise level, the analysis also includes the impact of the specific magnetization methods on the waveform amplitude.

**Figure 11:**
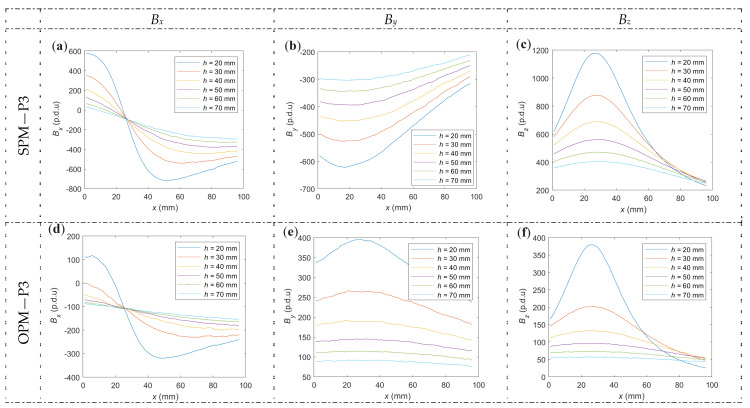

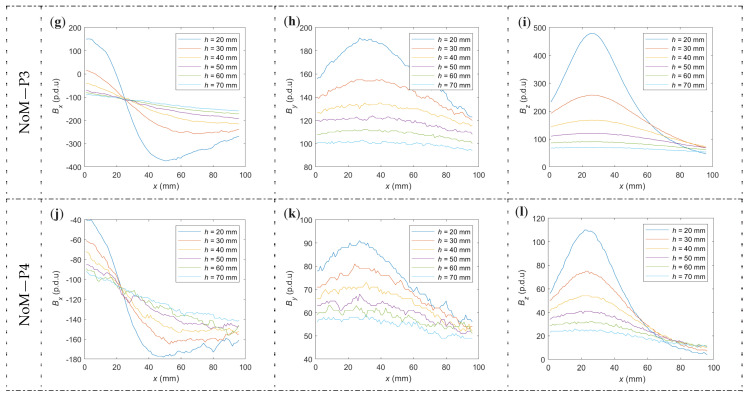
The measurements of spatial components of magnetic induction vs. *x* position for six different concrete cover thicknesses, different samples, and magnetization methods. (**a**) *B_x_*, SPM, P4, (**b**) *B_y_*, SPM, P4, (**c**) *B_z_*, SPM, P4, (**d**) *B_x_*, OPM, P4, (**e**) *B_y_*, OPM, P4, (**f**) *B_z_*, OPM, P4, (**g**) *B_x_*, NoM, P4, (**h**) *B_y_*, NoM, P4, (**i**) *B_z_*, NoM, P4, (**j**) *B_x_*, NoM, P3, (**k**) *B_y_*, NoM, P3, (**l**) *B_z_*, NoM, P3.

In electromagnetic and magnetic tests of RC structures, the tested samples are usually scanned in the direction perpendicular to the reinforcing bar (in the *x*-axis). Therefore, the noise level analysis is conducted based on the components of magnetic induction (*B_x_*, *B_y_*, *B_z_*) as a function of the transducer position *x*. The examples of measured waveforms are presented in Figure 11 (the presented results refer to samples P3 and P4).

Results shown in Figure 11 prove that it is possible to detect reinforcing bars (rebars) and even identify the parameters of an RC structure in the case of no magnetization (NoM). However, this possibility comes with several problems. Each rebar possesses residual magnetization. However, in the process of production, transport, construction, or exploitation, rebars may become magnetized to some degree. Therefore, in the case of NoM, it is impossible to preliminary determine how rebars are magnetized.

**Figure 12:**
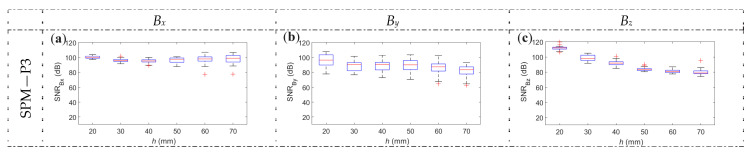

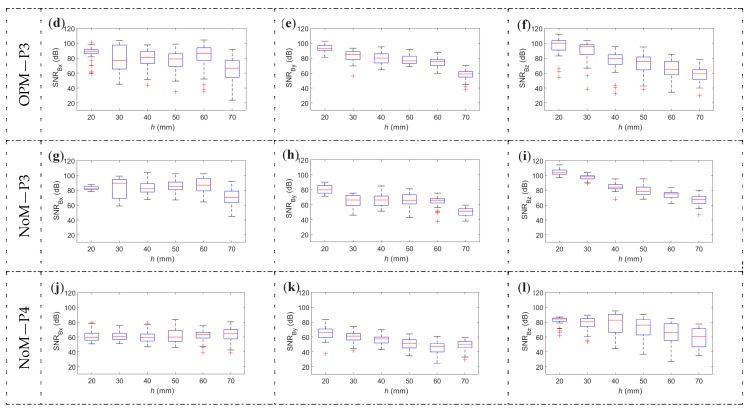
The SNR (signal to noise ratio) calculated for spatial components of magnetic induction vs. *h* (concrete cover thickness), for 31 measurements made in the measurement range +/− 150 mm from central measurement, (**a**) *B_x_*, SPM, P4, (**b**) *B_y_*, SPM, P4, (**c**) *B_z_*, SPM, P4, (**d**) *B_x_*, OPM, P4, (**e**) *B_y_*, OPM, P4, (**f**) *B_z_*, OPM, P4, (**g**) *B_x_*, NoM, P4, (**h**) *B_y_*, NoM, P4, (**i**) *B_z_*, NoM, P4, (**j**) *B_x_*, NoM, P3, (**k**) *B_y_*, NoM, P3, (**l**) *B_z_*, NoM, P3.

An example is shown in Figure 11g–l, where the P3 rebar possesses a relatively strong magnetic field, while the magnetic field of the P4 rebar is much weaker. In this case, the waveform amplitude and relation between spatial components cannot be used in the identification process. Another problem with NoM is noise. The weaker the signal, the greater the influence of noise. In the absence of magnetization, the noise level is noticeably higher than in the cases of SPM or OPM. The quantitative noise analysis is shown in Figure 12. The main advantage of using any form of magnetization is the possibility of obtaining predictable results (amplitude, noise level, etc.).

Moreover, it is observed that the energy of the received signal is higher in the case of SPM than in OPM. The high amplitude value allows for significantly improved SNR (signal-to-noise ratio), as shown in Figure 12.

Figure 12 graphically presents the SNR statistical analysis obtained for different concrete cover thicknesses, spatial components, and magnetization methods. The *x*-scans used to calculate the statistics are taken in the middle of the sample (*y* = 200 mm) and at thirty-one other *y*-axis transducer positions. Starting in *y* = 50 mm position (Figure 3). Then, the transducer was moved in ten-millimeter steps to the *y* = 350 mm position (central position ±150 mm with step of 10 mm). The statistics presented in Figure 12 do not consider waveform deformation but only noise. The used Butterworth filter eliminates only high-frequencies components (noise). The aspect of deformations is considered in the further part of this work.

The lowest impact of the noise is observed in the case of SPM. It is caused by both lower noise and higher waveform energy. The next advantage of this magnetization method is the very high repeatability (also in the case of SNR). Moreover, in contrast to NoM and OPM in the case of SPM, there is also no significant decrease in the indicator (SNR) value for larger concrete cover thicknesses, which the greater amplitude of the waveform may cause.

OPM magnetization, similar to SPM, makes the results predictable. However, the impact of the magnetization is weaker in the case of OPM. As a result, both the noise level and number of outliers are larger. These problems can be corrected to some extent by using stronger magnets. However, the use of stronger magnets can cause other problems, such as distortion caused by the direct impact of the magnet on the sensor.

#### 3.2.2. Separation of the Database Classes

Separation (distance between database classes) is one of the basic parameters that should be examined when comparing magnetization methods. This parameter directly impacts the effectiveness and simplicity of identification (classifying the measurement into a specific class). For vector measurements (in three-dimensional space), Formula (2) (for the distance between database classes) takes the form of (4):

(4) $$d_{a}\left(\alpha ,\beta \right)=\sqrt{{\left(\alpha_{x}-\beta_{x}\right)}^{2}+{\left(\alpha_{y}-\beta_{y}\right)}^{2}+{\left(\alpha_{z}-\beta_{z}\right)}^{2}}$$

where α = *B*_max_{Pi} and β = *B*_max_{Pj}; *B*_max_ is the maximal value of the waveform of magnetic induction, calculated as the difference between the largest and smallest waveform values in the case of *B_x_*, and as the largest of the absolute value in the case of *B_y_* and *B_z_*; {Pi} and {Pj} elements show for which sample type of the set {P1, P2, P3, P4} measurement was made (waveform is taken); and *d*_a_ is a distance between α = *B*_max_{Pi} and β = *B*_max_{Pj}.

**Figure 13:**
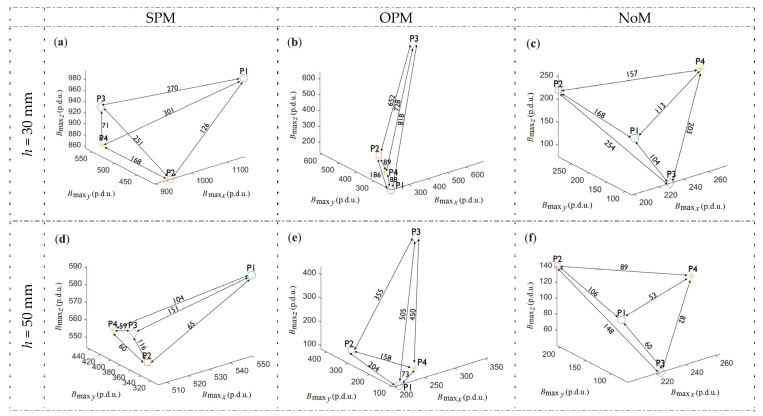
Euclidean distance between the amplitudes received for samples (P1–P4), three types of magnetization, and two examples of concrete cover thicknesses. (**a**) *h* = 30 mm, SPM, (**b**) *h* = 30 mm, OPM, (**c**) *h* = 30 mm, NoM, (**d**) *h* = 50 mm, SPM, (**e**) *h* = 50 mm, OPM, (**f**) *h* = 30 mm, NoM.

Separations between the classes (all kinds of rebars, six different *h*) are calculated according to (4) and shown in Figure 13 and in Table 3.

Based on Figure 13 and Table 3, it is impossible to select a better magnetization method due to database class separations. Different methods perform better in different cases. At higher values of *h*, separation is usually higher in the case of OPM. However, even then, it is not unambiguous.

Three types of reinforcing bars are used during the tests. Relatively soft and flexible AI class steel (sample P1), and two types of hard steel: AIII (sample P4) and AIIIN (samples P2 and P3). In the case of SPM magnetization, the result of measurement on P1 is strongly separated from other measurements. Similarly, in the case of OPM magnetization, the P4 measurement is strongly separated. Without magnetization (NoM), the measurement separation is accidental and results mainly from the sample’s residual magnetization level. Therefore, in the case of NoM, the separation (even if it is high) cannot be considered as an advantage.

In order to make the separation parameter independent of the amplitude, Formula (4) is modified to the form of (5):

(5) $$d_{r}\left(\alpha ,\beta \right)=\sqrt{\frac{{\left(\alpha_{x}-\beta_{x}\right)}^{2}}{{\left(\left(\alpha_{x}+\beta_{x}\right)/2\right)}^{2}}+\frac{{\left(\alpha_{y}-\beta_{y}\right)}^{2}}{{\left(\left(\alpha_{y}+\beta_{y}\right)/2\right)}^{2}}+\frac{{\left(\alpha_{z}-\beta_{z}\right)}^{2}}{{\left(\left(\alpha_{z}+\beta_{z}\right)/2\right)}^{2}}}\times 100\%$$

where α = *B*_max_{Pi} and β = *B*_max_{Pj}; *B*_max_ is the maximal value of the waveform of magnetic induction, calculated as the difference between the largest and smallest waveform values in the case of *B_x_*, and as the largest of the absolute value in the case of *B_y_* and *B_z_*; {Pi} and {Pj} elements show for which sample type of the set {P1, P2, P3, P4} measurement was made (waveform is taken); and *d*_a_ is a distance between α = *B*_max_{Pi} and β = *B*_max_{Pj}.

The results obtained based on the calculations using Equation (5) are presented in Table 4.

Table 4 shows that the relative separation is several times greater in the OPM case (relative to SPM). It is a significant difference from Table 3. The advantage of OPM in this area increases even more, together with the increase in the concrete cover thickness.

### 3.3. Impact of Magnetization on the Measurements Made along y-Axis

Scanning in the *y* direction (along the rebar) is not often used (unless it detects damage to the rebar itself) in the identification of the RC structure parameters process. However, the tests showed that *y*-scans could be used for this purpose. The second part of the subsection tests how a strong magnetic field may affect the results if the sensor is moved too close to the magnets. Two issues are investigated in this topic. The measurement area in which *x*-direction scans can be carried out without distortions and to what extent the change in the position of the sensor on the *y*-axis affects the *x*-scans. The results of these studies may be helpful in area testing and in the selection of magnet strength.

#### 3.3.1. Scans in *y*-Direction

**Figure 14:**
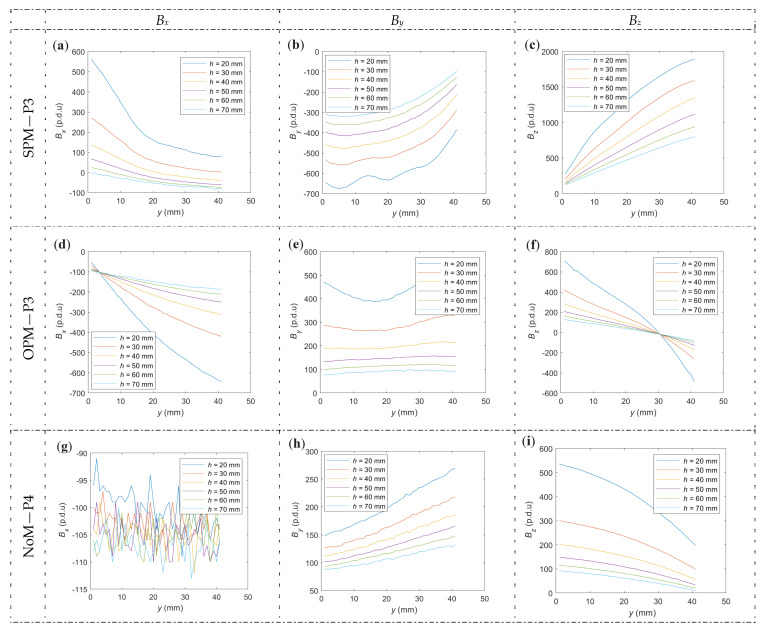
Scan in the *y*-direction, three types of magnetization, and three spatial components. (**a**) SPM, *B_x_*, (**b**) SPM, *B_y_*, (**c**) SPM, *B_z_*, (**d**) OPM, *B_x_*, (**e**) OPM, *B_y_*, (**f**) SPM, *B_z_*, (**g**) NoM, *B_x_*, (**h**) NoM, *B_y_*, (**i**) NoM, *B_z_*.

In order to identify the RC parameters of the structure, scans in the *x*-direction (*x*-scan) and the *y*-direction (*y*-scan) can be used. Exemplary results are shown in Figure 14.

The curves shown in Figure 14 are regular and resemble an arc or a straight line. The angle of inclination and the offset of the waveforms change with the change in concrete cover thickness. Measurements are very repeatable and regular. Theoretically, it is possible to identify structure parameters based on *y*-scans. However, such identification would be more complicated than in the case of scans along the *x*-axis. Measurements of this type are strongly affected by a large number of variables, including: concrete cover thickness, diameter and class of the rebar. Even a slight asymmetry in magnetization (location or unequal strength of the magnet fields) can make a significant difference. Scans in the *x*-direction are much more resistant to such asymmetry. Therefore, they are also easier to interpret and analyze.

#### 3.3.2. Scanning Area and Area Testing Sensors

**Figure 15:**
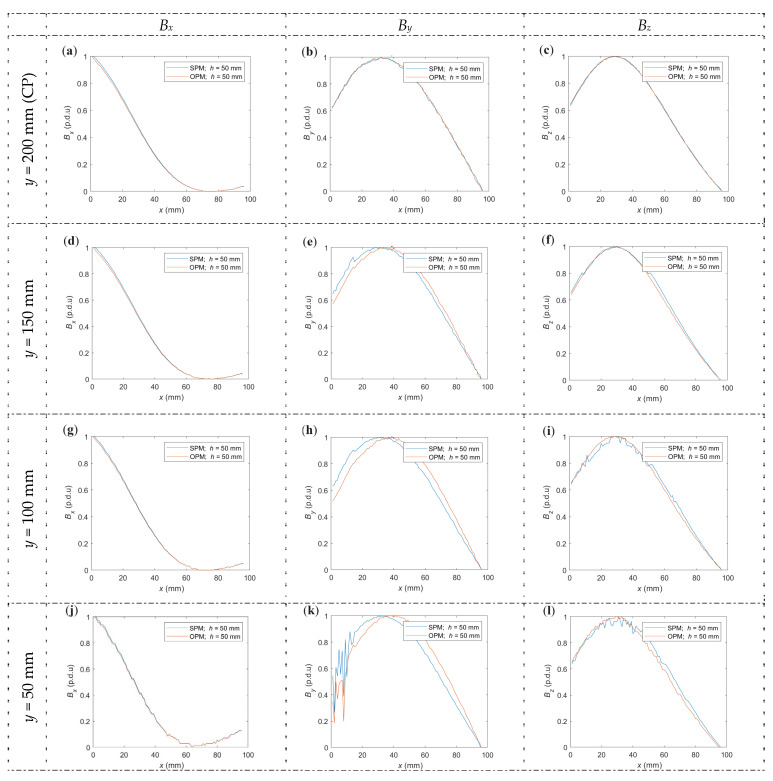

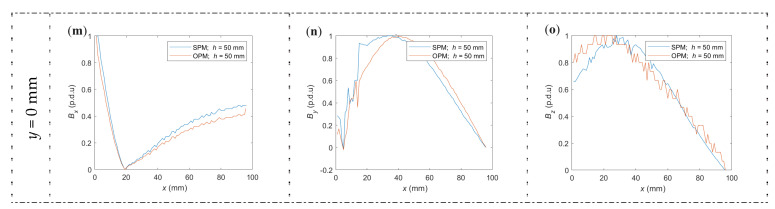
Normalized measurements in the *x*-axis shifted in the *y*-axis from the center position, two magnetization methods. (**a**) *B_x_*, central point (*y* = 200 mm), (**b**) *B_y_*, central point (*y* = 200 mm), (**c**) *B_z_*, central point(*y* = 200 mm), (**d**) *B_x_*, *y* = 150 mm, (**e**) *B_y_*, *y* = 150 mm, (**f**) *B_z_*, *y* = 150 mm, (**g**) *B_x_*, *y* = 100 mm, (**h**) *B_y_*, *y* = 100 mm, (**i**) *B_z_*, *y* = 100 mm, (**j**) *B_x_*, *y* = 50 mm, (**k**) *B_y_*, *y* = 50 mm, (**l**) *B_z_*, *y* = 50 mm, (**m**) *B_x_*, *y* = 0 mm, (**n**) *B_y_*, *y* = 0 mm, (**o**) *B_z_*, *y* = 0 mm.

The *y*-coordinate position, where the *x*-scan is made, makes a difference and is essential in at least three cases. The first is the design of transducers for area testing. Such transducers may consist of a matrix of sensors arranged in the XY plane. The change of the magnetic field relative to the position on the *y*-axis is an essential issue for such sensors because it allows us to make corrections for differences resulting from different sensor positions. The research can also be utilized to test the susceptibility of measurements to magnetization asymmetry. The third case is that the experiments also showed a particular measurement area (along the *y*-axis) where the scans along the *x*-axis are similar and only slightly differ in shape and amplitude. After exceeding this range, the direct influence of the magnets on the sensor becomes dominant, and the waveforms obtained during scans along the *x*-axis significantly change their shape. Figure 15 shows how the shape of the waveform changes by moving the sensor by a certain distance along the *y*-axis. The figure shows the shifts to the left by 0, 50, 100, 150, and 200 mm relative to the central point (CP; *y* = 200 mm) in the *y*-axis.

The magnetization method affects the amplitude and offset, but it does not affect the shape of the waveforms obtained from scans along the *x*-axis (if measurements are made in the middle of the measurement area). The study showed very high reproducibility of the results. The waveforms shown in Figure 15 are received for different methods of magnetization (OPM and SPM) at *h* = 50 mm. At lower values of concrete cover thickness, the waveforms covered each other, which made the graph illegible.

**Figure 16:**
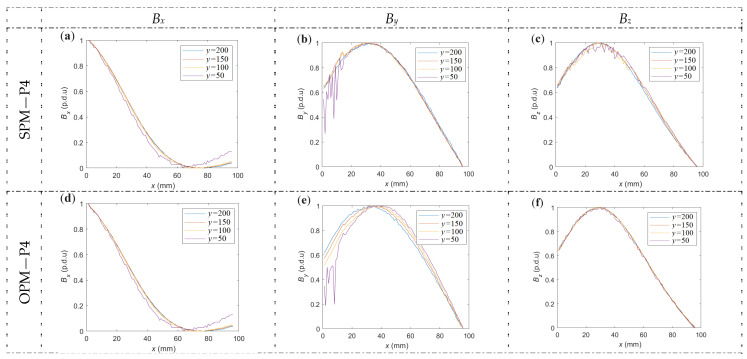
Comparison of the shapes of normalized waveforms measured in different *y*-coordinates, four different *y*-coordinates, and two methods of magnetization. (**a**) *B_x_*, SPM, (**b**) *B_y_*, SPM, (**c**) *B_z_*, SPM, (**d**) *B_x_*, OPM, (**e**) *B_y_*, OPM, (**f**) *B_z_*, OPM. *y* = 200 mm is a central point *y*-coordinate.

Magnetization can cause deformations of the waveform shape. In the case of OPM magnetization (spatial component *B_y_*), the deformation increases together with the shift. This effect is shown more clearly in Figure 16. In the case of SPM magnetization, such deformation is not observed (within the measurement area). Moreover, together with the shift, the noise also increases (both OPM and SPM magnetization—effect visible mostly for *B_y_* and *B_z_*). After moving the sensor by 200 mm (*y* = 0 mm) relative to the central point, the shape of the waveforms begins to deform strongly (both magnetization methods). It can be assumed that the limit of the measuring area is about ±150 mm from the central point (*y* between 50 and 350 mm).

Figure 16 shows that clear deformations appearing at a shift of 150 mm (*y* = 50 mm) and 140 mm (*y* = 60 mm) did not occur yet.

### 3.4. Impact of Magnetization on the Measurements Made along z-Axis

This subsection analyzes the dependence of the *B*_max_ as a function of position on the axis *z*. Scanning in the *z* direction is not often used, but it has one significant advantage over other scans. The curve’s shape is independent of the method of magnetization (all of them: NoM, OPM, SPM) and also of the class and diameter of the rebar. Therefore, this function best identifies the *h* value (in the multivariable problems).

**Figure 17:**
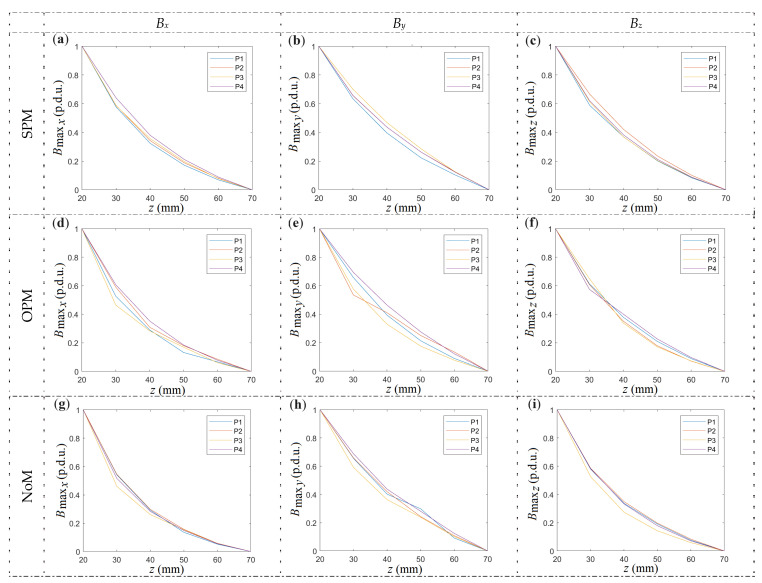
Summary of normalized *B*_max_ = *f*(*z*) curves for different samples (P1–P4), methods of magnetization, and spatial components of magnetic induction. (**a**) *B_x_*, SPM, (**b**) *B_y_*, SPM, (**c**) *B_z_*, SPM, (**d**) *B_x_*, OPM, (**e**) *B_y_*, OPM, (**f**) *B_z_*, OPM, (**g**) *B_x_*, NoM, (**h**) *B_y_*, NoM, (**i**) *B_z_*, NoM.

The *B*_max_ in function of *z*-axis position curves obtained for different samples (P1–P4) differ in amplitude (gain) and offset, but their shape is practically the same, as shown in Figure 17.

**Figure 18:**
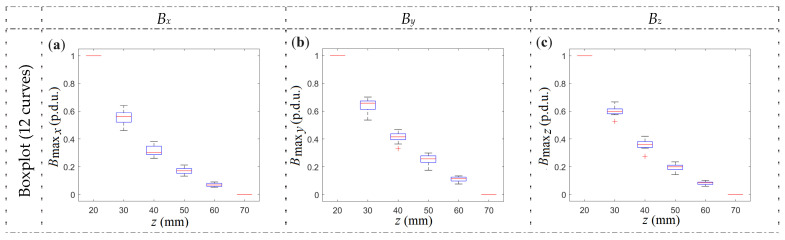
Statistical summary of normalized magnetic induction (*B*_max_) curves for different samples (P1–P4), methods of magnetization, and spatial components of magnetic induction: (**a**) *B_x_*, (**b**) *B_y_*, (**c**) *B_z_*.

Based on Figure 17, it can be observed that the most minor differences between samples appear in the case of SPM. It is worth noting that the shape of the curve does not change significantly regardless of the method of magnetization. A statistical summary of the shape of the curves for all samples and methods of magnetization (12 curves for each figure; four samples × three magnetization methods) is shown in Figure 18.

**Figure 19:**
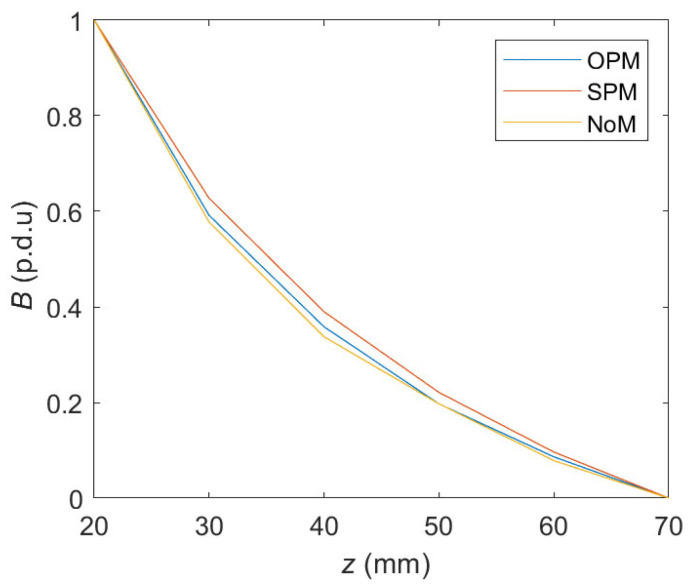
The average value of the normalized curve *B*_max_ (number of included cases: three spatial components of magnetic induction × four samples).

Using *z*-scans allows the *h* parameter identification to be completely independent not only of the method of magnetization (OPM, SPM, NoM, which is presented in Figure 19) but also of the diameter and class of the rebar. It would greatly simplify the classification process. In addition, such a measurement also provides amplitude (gain) and offset values for each sample. These parameters can be used to identify the rebar’s diameter and class. Even taking into account very different cases (including those ineffective, such as NoM), the obtained variation of the results is minimal (Figure 18) and allows the identification of the cover thickness with an accuracy of a few millimeters (in the worst case).

## 4. Conclusions

Magnetization (any type) in magnetic measurements makes the measurements predictable. It allows the use of amplitude and offset attributes in analysis and identification processes (in the absence of magnetization, these parameters assume random values, and then identification can be based only on shape attributes).

Experiments show that higher predictability is achieved with opposite pole magnetization (OPM) than with the same pole magnetization (SPM).

Changing the typical OPM magnetization to SPM significantly increases the signal amplitude, repeatability, and signal-to-noise ratio. To a certain point, SPM magnetization (unlike OPM) also does not cause waveform deformation in cases where the measurement is shifted from the central point in the *y*-axis.

The study also shows that the function of the amplitude in the *z*-position domain (distance between the transducer and a tested object—the rebar in this case) is practically independent of the type of the tested rebar or the method of magnetization. Therefore, using the *z*-scan can be the best way to identify concrete cover thickness (in cases where multiple parameters are identified simultaneously).

Tests prove that in the case of an *x*-scan (as in the case of scans on the *z*-axis), the magnetization method impacts the amplitude and offset of the waveform but does not affect the shape of the waveform. The shape of the normalized waveforms along any axis depends only on the position of the sensor relative to the rebar (distance and angle between them) in each measurement position that creates this waveform. The amplitude and offset are influenced by many factors, including the strength of magnets, class of reinforcing steel, place of measurement, asymmetry, etc.

The simulations presented in this work confirm the measurement results’ correctness and indicate the importance of the material’s magnetic permeability. In simple terms, the harder and less flexible the steel from which the reinforcing bars are made, the stronger the signal received from the magnetic sensor (class identification).

## Figures and Tables

**Table 1:** A comparison of RMT and CMT properties.

RMT	CMT
− The simplicity of the system;	− More complex system;
− No excitation;	− An excitation system is required;
− Level of residual magnetization is unpredictable and can change in time	− High repeatability and predictability of results
− Relatively weak signal;	− Signal energy can be modulated;
− Low noise resistance;	− Higher noise resistance;
− No control.	− The ability to influence the received signal through the excitation system.

**Table 2:** A comparison of magnetic, capacitive, and EC methods.

Factor	Capacitive	EC	Magnetic
Resolution	•••	••	•
Range	•	••	••
Low cost	•••	••	•••
Area testing (multisensory transducer)	••	•	•••
Work in dirty environments	-	••	••
Work with thin materials	••	•	••
Material versatility	••	•	•
Simplicity of probe mounting	•••	••	•••
Bandwidth	•	••	-

‘-’ not applicable; ‘•’ worst; ‘••’ intermediate; ‘•••’ best.

**Table 3:** Comparison of separations (distances *d*_a_) between the amplitudes (absolute values) received for samples (P1–P4), for three types of magnetization, and six *h* values.

*h* (mm)	20			30			40			50			60			70		
Excitation	SP	OP	No	SP	OP	No	SP	OP	No	SP	OP	No	SP	OP	No	SP	OP	No
*d_a_*(P1,P2)	549	276	214	251	186	168	132	192	142	65	204	115	38	218	106	39	223	92
*d_a_*(P1,P3)	565	1324	183	270	818	104	184	642	83	151	505	73	135	413	60	126	353	52
*d_a_*(P1,P4)	693	276	394	301	98	113	173	79	62	104	73	64	71	65	53	63	61	45
*d_a_*(P2,P3)	235	1048	332	168	652	254	132	495	207	114	355	173	101	259	148	90	203	127
*d_a_*(P2,P4)	199	100	344	126	89	157	86	138	120	60	158	105	38	168	89	27	172	77
*d_a_*(P3,P4)	217	1064	432	71	728	203	56	570	134	59	450	105	74	368	82	80	316	67

SP—same pole magnetization (SPM); OP—opposite pole magnetization (OPM); No—no magnetization (NoM). 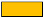 highest separation for a given *h*; 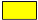 medium separation for a given *h*.

**Table 4:** Comparison of relative separations (distances *d*_r_) between the amplitudes received for samples (P1–P4) for three types of magnetization and six *h* values.

*h* (mm)	20			30			40			50			60			70		
Excitation	SP	OP	No	SP	OP	No	SP	OP	No	SP	OP	No	SP	OP	No	SP	OP	No
*d_r_*(P1,P2)	37	94	83	25	83	81	19	97	83	14	117	77	13	138	83	14	156	81
*d_r_*(P1,P3)	45	213	99	38	197	89	38	196	90	39	194	96	40	197	97	41	205	100
*d_r_*(P1,P4)	51	90	108	34	50	53	32	47	39	27	52	36	23	66	38	22	86	39
*d_r_*(P2,P3)	35	150	152	33	140	150	30	136	150	29	120	150	28	109	151	28	103	151
*d_r_*(P2,P4)	29	27	96	26	35	70	21	64	62	17	81	59	11	94	58	8	107	60
*d_r_*(P3,P4)	18	152	150	10	165	128	11	165	120	13	164	116	19	166	112	23	172	107

SP—same pole magnetization (SPM); OP—opposite pole magnetization (OPM); No—no magnetization (NoM). 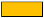 highest separation; 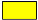 medium separation.

## Data Availability

Data available on request.

## References

[1] Neville A.M. (2011). Properties of Concrete.

[2] Li V.N., Demina L.S., Vlasenko S.A., Tryapkin E.Y. (2020). Assessment of the Impact of the electromagnetic field of the catenary system on crack formation in reinforced concrete supports. IOP Conf. Ser. Mater. Sci. Eng..

[3] Li V., Demina L., Vlasenko S. (2020). Assessment of the concrete part of the contact system supports in the field. E3S Web Conf..

[4] Maierhofer C., Reinhardt H.W., Dobmann G. (2010). Non-Destructive Evaluation of Reinforced Concrete Structures.

[5] Dadras A., Ghaderiaram A., Fotouhi M., Schlangen E. (2022). A Review on Non-Destructive Evaluation of Civil Structures Using Magnetic Sensors. 10th European Workshop on Structural Health Monitoring (EWSHM 2022).

[6] Frankowski P.K., Chady T., Zielinski A. (2021). Magnetic force induced vibration evaluation (M5) method for frequency analysis of rebar-debonding in reinforced concrete. Measurement.

[7] Frankowski P.K., Chady T. (2022). Impact of Magnetization on the Evaluation of Reinforced Concrete Structures Using DC Magnetic Methods. Materials.

[8] Frankowski P.K., Chady T. (2023). Evaluation of Reinforced Concrete Structures with Magnetic Method and ACO (Amplitude-Correlation-Offset) Decomposition. Materials.

[9] Gobov Y.L., Mikhailov A.V., Smorodinskii Y.G. (2018). Magnetic Method for Non-destructive Testing of Rebar in Concrete. Russ. J. Nondestruct. Test..

[10] Zhou J., Qiu K., Deng B., Zhang G., Ye G. (2022). A NDT Method for Location and Buried Depth Measurement of Rebars in Concrete Pole. IEEE Trans. Instrum. Meas..

[11] Perin D., Göktepe M. (2012). Inspection of rebars in concrete blocks. Int. J. Appl. Electro-Magn. Mech..

[12] Lo C.C.H., Nakagawa N. (2013). Evaluation of eddy current and magnetic techniques for inspecting rebars in bridge barrier rails. AIP Conf. Proc..

[13] Mosharafi M., Mahbaz S., Dusseault M., Vanheeghe P. (2020). Magnetic detection of corroded steel rebar: Reality and simulations. NDT E Int..

[14] Diogenes A.G., Moura E.P., Machado A.S., Gonçalves L.L. (2022). Corrosion evaluation of carbon steel bars by magnetic non-destructive method. Non-Destr. Test. Eval..

[15] Junli Q., Weiping Z., Yue J. (2023). Quantitative linear correlation between self-magnetic flux leakage field varia-tion and corrosion unevenness of corroded rebars. Measurement.

[16] Mosharafi M., Mahbaz S.B., Dusseault D. (2020). Bridge deck assessment using infrastructure corrosion assessment magnetic method (iCAMM™) technology, a case study of a culvert in Markham city, Ontario, Canada. NDT E Int..

[17] Jiang S.H., Wang H., Liu A.Z. (2020). Rebar corrosion monitoring using magnetic gradient and partial modulus. Measurement.

[18] Scheel H., Hillemeier B. (1997). Capacity of the remanent magnetism method to detect fractures of steel in ten-dons embedded in prestressed concrete. NDT & E Int..

[19] Mosharafi M., Mahbaz S., Dusseault M.B. (2020). Magnetic Data Pattern Features at Longitudinal Defect Sites in Rebars Scanned by a Passive Magnetic Inspection Technology. J. Environ. Eng. Geophys..

[20] Kai T., Jianting Z., Ruiqiang Z., Wenxue H., Yinghao Q., Chongshen C. (2021). Experimental study on rebar stress measurement based on force-magnetic coupling under excited magnetic field. Measurement.

[21] Kai T., Jianting Z., Xiaotao M., Huajian Y., Ruiqiang Z. (2023). Investigation of the effect of initial magnetization state on the force-magnetic coupling effect of rebars. J. Magn. Magn. Mater..

[22] Jianting Z., Huajian Y., Kai T., Yinghao Q., Hong Z., Ruiqiang Z. (2023). Research on quantitative evaluation of rebar stress based on weak magnetic effect. J. Magn. Magn. Mater..

[23] Eslamlou A.D., Ghaderiaram A., Schlangen E., Fotouhi M. (2023). A review on non-destructive evaluation of construction materials and structures using magnetic sensors. Constr. Build. Mater..

[24] Ahmad M.I.M., Arifin A., Abdullah S., Jusoh W.Z.W., Singh S.S.K. (2015). Fatigue crack effect on magnetic flux leakage for A283 grade C steel. Steel Compos. Structuctures.

[25] Mahbaz S.B., Dusseault M.B., Cascante G., Vanheeghe P. (2017). Detecting defects in steel reinforcement using the passive magnetic inspection method. J. Environ. Eng. Geophys..

[26] Mordor Intelligence Magnetic Sensors Market Size & Share Analysis—Growth Trends & Forecasts (2023–2028). https://www.mordorintelligence.com/industry-reports/magnetic-sensor-market.

[27] Khan M.A., Sun J., Li B., Przybysz A., Kosel J. (2021). Magnetic sensors-A review and recent technologies. Eng. Res. Express.

[28] Djamal M., Ramli R. (2017). Giant Magnetoresistance Sensors Based on Ferrite Material and Its Applications. Magnetic Sensors—Development Trends and Applications.

[29] Yang S., Zhang J. (2021). Current Progress of Magnetoresistance Sensors. Chemosensors.

[30] Szymanik B., Frankowski P.K., Chady T., Chelliah C.R.A.J. (2016). Detection and Inspection of Steel Bars in Reinforced Concrete Structures Using Active Infrared Thermography with Microwave Excitation and Eddy Current Sensors. Sensors.

[31] Frankowski P.K., Sikora R., Chady T. (2016). Identification of rebars in a reinforced mesh using eddy current method. 42nd Annual Review of Progress in Quantitative Non-Destructive Evaluation.

[32] Drobiec Ł., Jasiński R., Mazur W. (2019). Accuracy of Eddy-Current and Radar Methods Used in Reinforcement Detection. Materials.

[33] Chady T., Frankowski P.K. (2013). Electromagnetic Evaluation of Reinforced Concrete Structure. Review of Progress in Quantitative Non-Destructive Evaluation.

[34] Frankowski P.K. Eddy current method for identification and analysis of reinforcement bars in concrete structures. Proceedings of the 2011 IEEE 3rd International Students Conference on Electrodynamics and Mechatronics (SCE III).

[35] Frankowski P.K., Sikora R., Chady T. (2016). Identification of rebars in a reinforced mesh using eddy current method. AIP Conf. Proc..

[36] Han X., Wang P., Cui D., Tawfik T.A., Chen Z., Tian L., Gao Y. (2023). Rebar corrosion detection in concrete based on capacitance principle. Measurement.

[37] Han X., Li G., Wang P., Chen Z., Cui D., Zhang H., Tian L., Zhou X., Jin Z., Zhao T. (2022). A new method and device for detecting rebars in concrete based on capacitance. Measurement.

[38] Zhang M., Ma L., Soleimani M. (2014). Magnetic induction tomography guided electrical capacitance tomography imaging with grounded conductors. Measurement.

[39] Solla M., Lagüela S., Fernández N., Garrido I. (2019). Assessing Rebar Corrosion through the Combination of Non-destructive GPR and IRT Methodologies. Remote Sens..

[40] Tešić K., Baricević A., Serdar M. (2021). Non-destructive Corrosion Inspection of Reinforced Concrete Using Ground-Penetrating Radar: A Review. Materials.

[41] Tosti F., Benedetto F., Munisami K., Sofroniou A., Alani A.M. A high-resolution velocity analysis to improve GPR data migration for rebars investigation. Proceedings of the EGU General Assembly 2018.

[42] Mechbal Z., Khamlichi A. (2017). Determination of concrete rebars characteristics by enhanced post-processing of GPR scan raw data. NDT E Int..

[43] Le T., Gibb S., Pham N., La H.M., Falk L., Berendsen T. Autonomous robotic system using non-destructive evaluation methods for bridge deck inspection. Proceedings of the 2017 IEEE International Conference on Robotics and Automation (ICRA).

[44] Xiang Z., Ou G., Rashidi A. (2021). Robust cascaded frequency filters to recognize rebar in GPR data with complex signal interference. Autom. Constr..

[45] Szymanik B., Chady T., Frankowski P.K. (2017). Inspection of reinforcement concrete structures with active infrared thermography. 43rd Annual Review of Progress in Quantitative Non-Destructive Evaluation.

[46] Keo S.A., Szymanik B., Le Roy C., Brachelet F., Defer D. (2023). Defect Detection in CFRP Concrete Reinforcement Using the Microwave Infrared Thermography (MIRT) Method—A Numerical Modeling and Experimental Approach. Appl. Sci..

[47] Tran H.Q. (2021). Passive and active infrared thermography techniques in non-destructive evaluation for concrete bridge. AIP Conf. Proc..

[48] Brachelet F., Keo S., Defer D., Breaban F. Detection of reinforcement bars in concrete slabs by infrared thermography and microwaves excitation. Proceedings of the QIRT 2014 Civil Engineering & Buildings.

[49] Teo S.A., Brachelet F., Defer D., Breaban F. (2022). Detection of Concrete Cover of Reinforcements in Reinforced Concrete Wall by Microwave Thermography with Transmission Approach. Appl. Sci..

[50] Garrido I., Solla M., Lagüela S., Rasol M. (2022). Review of InfraRed Thermography and Ground-Penetrating Radar Applications for Building Assessment. Adv. Civ. Eng..

[51] Mikhailov A.V., Gobov Y.L., Smorodinskii Y.G., Novoslugina A.P. (2019). Dipole model of magnetization of rebar in concrete. AIP Conf. Proc..

[52] Wegen G., Polder R.B., Breugel K. (2012). Guideline for service life design of structural concrete—A performance-based approach with regard to chloride induced corrosion. Heron.

[53] (2002). Concrete, Reinforced Concrete and Prestressed Structures. Static Calculations and Design.

[54] Johnson D.H. (2006). Signal-to-Noise Ratio. Scholarpedia.

[55] González R.C., Woods R.E. (2008). Digital Image Processing.

[56] Schafer R.W. (2011). What Is a Savitzky-Golay Filter? [Lecture Notes]. IEEE Signal Process. Mag..

